# Supplementary material for: Development of a Rapid Fluorescent Diagnostic System for Early Detection of the Highly Pathogenic Avian Influenza H5 Clade 2.3.4.4 Viruses in Chicken Stool
Source: Int J Mol Sci. 2022 Jun 4;23(11):6301. doi: 10.3390/ijms23116301 (PMC9181406; doi:10.3390/ijms23116301)
Supplement: Supplementary file 1 [file ijms-23-06301-s001.zip › ijms-1737403-supplementary.pdf]

Rapid Specific and Early Detection of Highly Emergency Pathogenic Avian Influenza H5 Subtype 2.3.4.4 Subclade in Chicken Feces

Supplementary Information

Supplementary Method

Supplementary tables

Table.S1. List of viruses in this study

Table.S2. Summary the result of clinical specimens testing

Supplementary figures

Figure.S1. Figure.S1. Expression of recombinant influenza H5/2.3.4.4b subclades by reverse genetics technology.

Figure S2. Designing of HA /H5N6 2.3.4.4c subclade globular head domain for antigen expression in E. coli systems.

Figure.S3. Production of mice monoclonal antibodies by Hybridoma technology.

Figure.S4. Establishment of rapid fluorescent immunochromatographic strip test (FICT) Europium nanoparticle–based.

Fig.S5. Measurement of KD values for antibodies by surface plasmon resonance (SPR) with Biacore T200 (GE Healthcare (Sweden) of WOOJUNG BIO, Inc Company.

Figure.S6. Raw fluorescence peaks from the test line (TL) and control line (CL) in FICT are showing for (n=3) for Optimization of lysis buffer for fluorescent immunochromatographic test (FICT) assay of Europium #11-4 and #23.3 strip coating.

Figure.S7. Raw fluorescence peaks from the test line (TL) and control line (CL) in FICT for testing of spiked samples.

Figure.S8. Raw of rRT-PCR for LoD in spiked samples.

Figure.S9. Specificity of the FICT assay.

Figure.S10. Detection of H5 virus by using Bionote Influenza Ag test kit.

Figure.S11. Determination of egg infective dose 50 (EID50) of virus stock.

Figure.S12. Raw data of FICT test to detect H9N2 and A(H5N6)/Duck/Foshan/41/2019 in clinical samples.

Fig.S13. Performance of RDT kit to detect H5N6 2.3.4.4c in clinical samples by using colloidal gold-based rapid diagnostic kit using the same pair mnAbs.

Supplementary Method

Measurement of binding affinities

Surface plasmon resonance (SPR) was used to analyze both binding affinities and kinetic parameters between peptide antibodies and H5N6 HA1 antigen by WOOJUNG BIO, Inc. (Suwon, Korea) using CM5 chip (GEHealthcare, Chicago, IL, USA).

Supplementary tables

Table. S1 List of viruses in this study

1	H1N1	A/H1N1/2009/CA
2	H3N2	Influenza A/H3N2 /2014 (RKBPV-VR-85)
3	H5N3	A/spot-billed duck/Korea/KNU SYG06/2006(H5N3)
4	H7N1	A/common teal/Korea/KNU YSR12/2012(H7N1)
5	H7N7	A/mallard/Korea/KNU GPH12/2011(H7N7)
6	H9N2	A/chicken/Korea/KNUSWR09/2009(H9N2)
7	H5/2.3.4.4b RGV	A/*Anas platyrhynchos*/Korea/W612/2017(H5N6)
8	H5/2.3.4.4c RGV	A/mallard/Korea/W452/2014(H5N8)
9	H5N6	A(H5N6)/Duck/Foshan/41/2019
10	H2N9	A/wild duck/South Korea/KNU18-102/2018(H2N9)

Table.S2. Summary the result of clinical specimens testing. The Cut-off Values of FICT were decided by highest ratio of TL per CL in normal group; cut off > 1.56; 0.3 for positive samples in feces and cloacal sample, respectively. "+", positive; "-", Negative; "N", Negative. "TL/CL", ratio of test line/control line.

Clinical specimens	Virus infection group	2 day post infection				
		Nunber of colection sample	rt PCR (Ct)	FICT TL/CL	FICT Result cut off > 1.56	RDT base gold colloid
Feces samples	None-infected (Normal)	1	N	0	-	-
		2	N	1.56	-	-
		3	N	0.32	-	-
		4	N	0.45	-	-
		5	N	0.29	-	-
	H9N2	1	25.30	0.00	-	-
		2	23.38	0.00	-	-
		3	N	0.00	-	-
	A/Duck/Foshan/41/2019 (H5N6) 2.3.4.4c	1	25.89	58.57	+	+
		2	29.31	18.4	+	+
		3	28.6	5.71	+	-
**Clinical specimens**	**Virus infection group**	**2 day post infection**				
		**Nunber of colection sample**	**rt PCR (Ct)**	**FICT TL/CL**	**FICT Result cut off > 0.3**	**RDT base gold colloid**
Cloacal samples	None-infected (Normal)	1	N	0.3	-	-
		2	N	0.02	-	-
		3	N	0.07	-	-
	H9N2	1	29.61	0.00	-	-
		2	31.91	0.00	-	-
		3	33.92	0.00	-	-
	A/Duck/Foshan/41/2019 (H5N6) 2.3.4.4c	1	30.51	0.08	-	-
		2	N	0	-	-
		3	27.52	0.06	-	-
Control	H5N6 (Postive control)		20.81			
	DW (Neg Ctrl)		N			

Supplementary figures

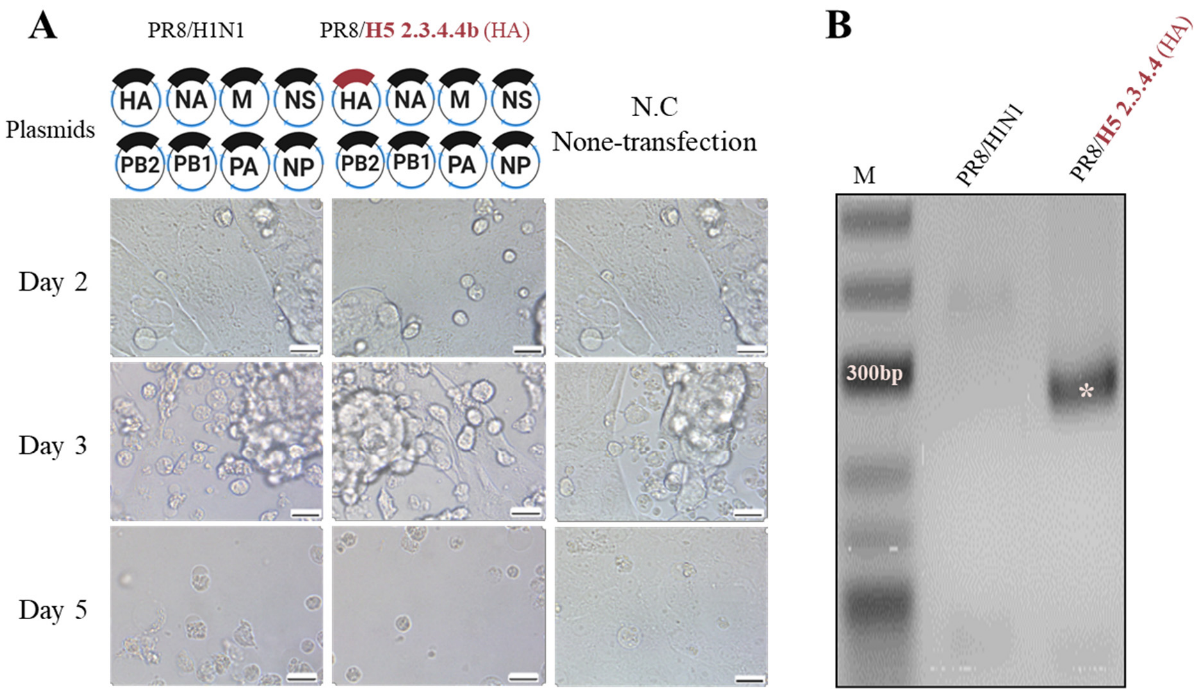

Figure.S1. Expression of recombinant influenza H5/2.3.4.4b subclades by reverse genetics technology. (A), Transfection of plasmids into 293T and MDCK cells (scale bar, 20μm;

original magnification, 400×). (B), confirmation of RGV by PCR with specific H5 subtypes primer. Specific band was obtained at 245 base pair.

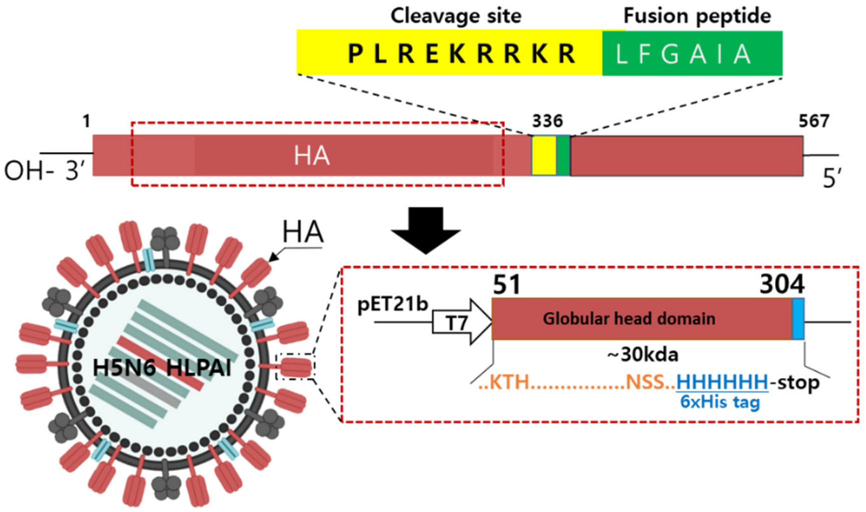

Figure S2. Designing of HA /H5N6 2.3.4.4c subclade globular head domain for antigen expression *in E. coli* systems.

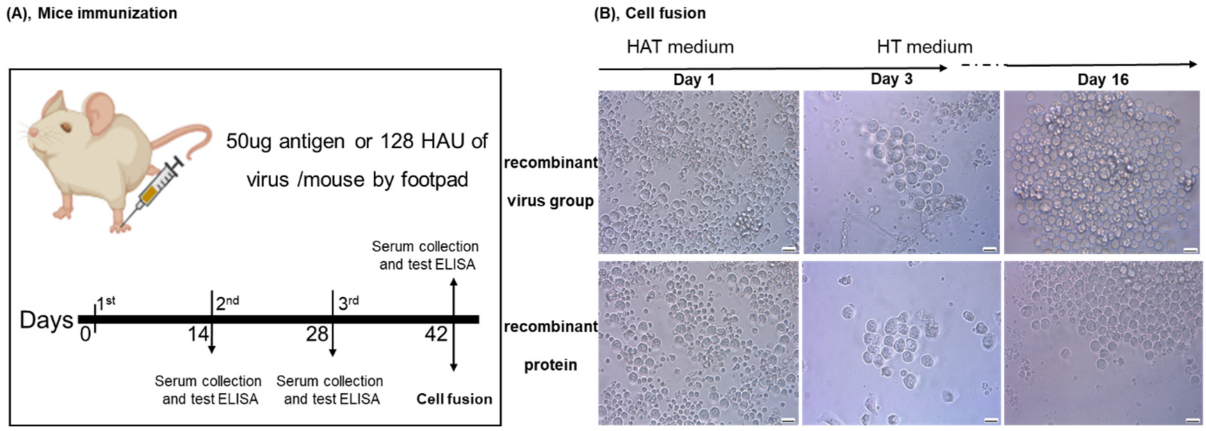

Figure.S3. Production of mice monoclonal antibodies by Hybridoma technology. (A) mouse was immunized by footpad method with 50ug recombinant protein or 128 HAU of recombinant virus for thrice times. (B), Selection of Hybridoma cell under HAT and HT medium for 16 days, the picture taken with original magnification, 400× and scale bar, 20µm.

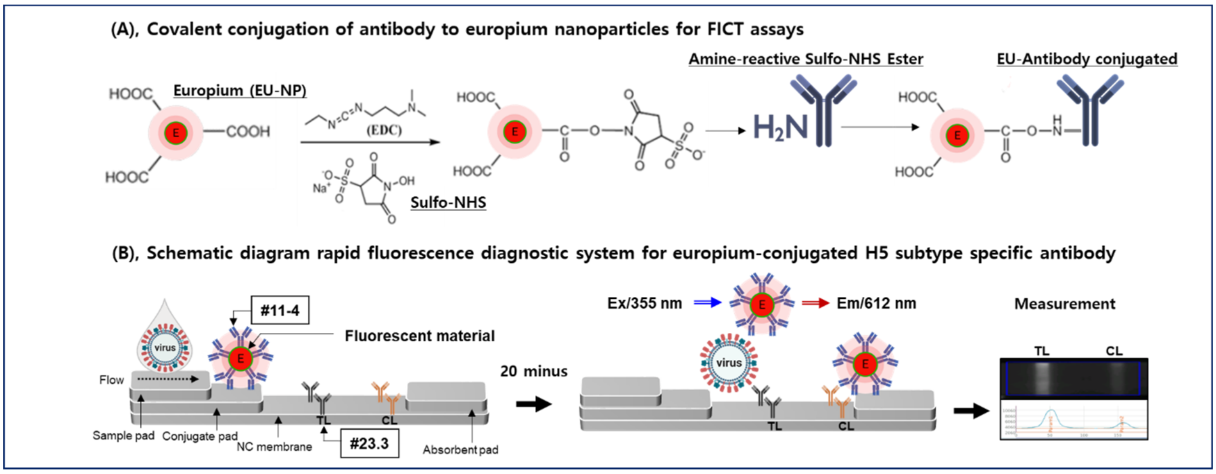

Figure.S4. Establishment of rapid fluorescent immunochromatographic strip test (FICT) Europium nanoparticle–based. (A), the EDC reactivated with the carboxyl group on the surface of nanoparticles to make a cross linker. In the presence of Sulfo-NHS EDC, it is create a more stable amine-reactive intermediate, which can bind to the amines group of antibody. (B), the antibody #11.4 conjugated to europium, #23.3 coated on strip with (3mg/ml) at test line. Control line coated with anti-mouse IgG (0.5mg/ml).

(A), Summary data of Measurement of KD values for antibodies.

Ligand	Analyte	Conc.	ka (1/Ms)	kd (1/s)	KD (M)	Rmax (RU)	Chi² (RU²)
H5N6-HA1	#9.1	0.488, 0.977, 1.953, 3.906, 7.813, 15.63, 31.25, 62.5, 125, 250 nM	1.953E+05	2.425E-05	1.242E-10	20.82	0.914
	#23.3	62.5, 125, 250, 500, 1000, 2000, 4000 nM	5.653E+03	3.315E-04	5.864E-08	9.844	0.174
	#11-4	62.5, 125, 250, 500, 1000, 2000, 4000 nM	9.769E+03	7.397E-06	7.572E-10	4.699	0.183

- K_D(Equilibrium dissociation rate constant), k_a (association rate constant), k_d(dissociation rate constant)

(B), RAW data.

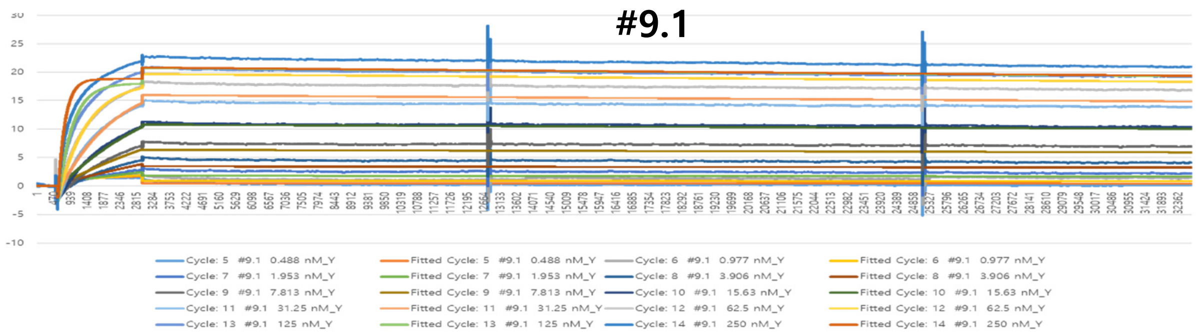

#23.3

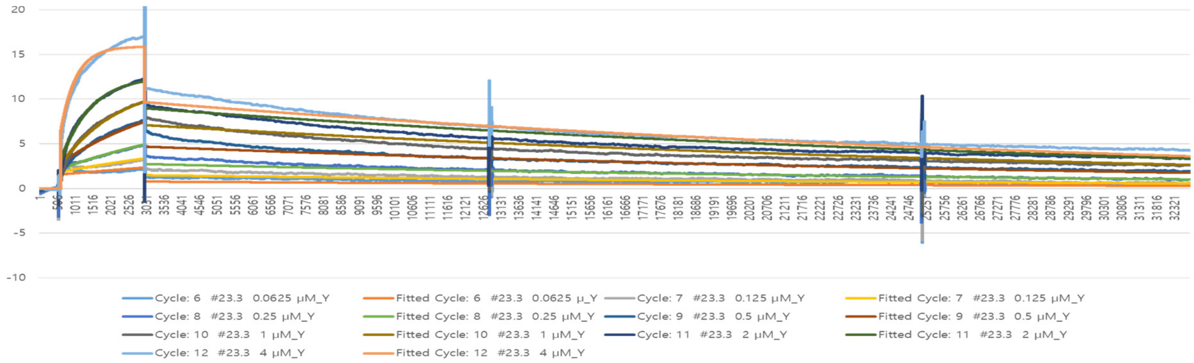

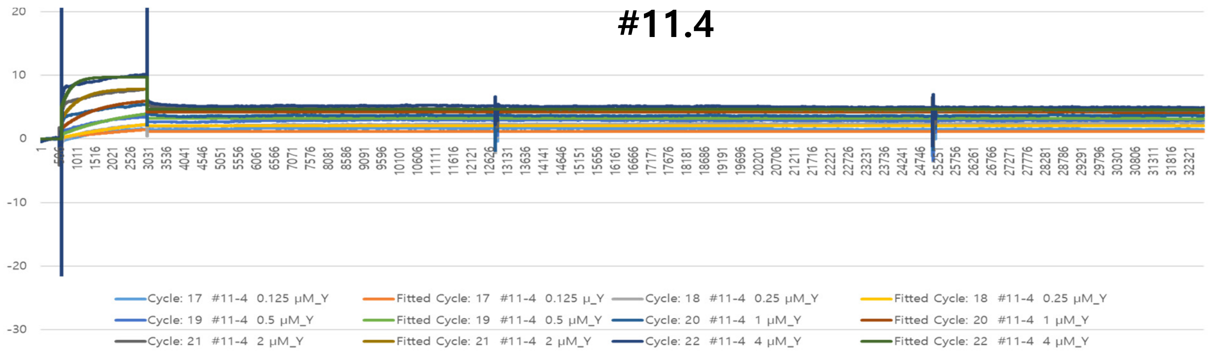

Fig. S5. Measurement of KD values for antibodies by surface Plasmon resonance (SPR) with Biacore T200 (GE Healthcare (Sweden) of WOOJUNG BIO, Inc. Company.

(A). pH6, 0.3% SDS.

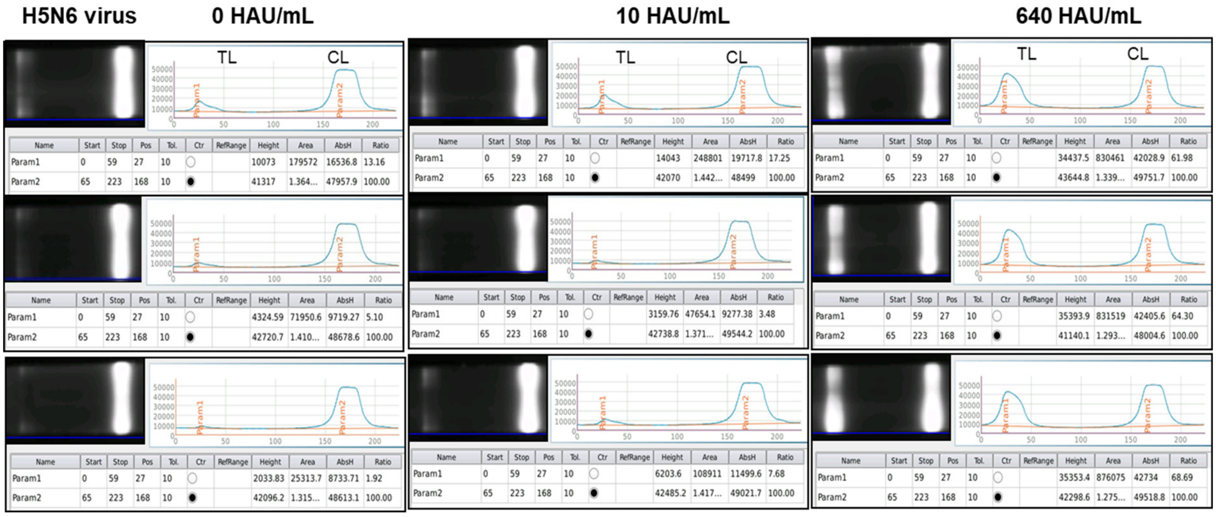

0 HAU/mL

Name	Start	Stop	Pos	Tol.	Ctr	RefRange	Height	Area	AbsH	Ratio
Param1	0	59	27	10	○		10073	179572	16536.8	13.16
Param2	65	223	168	10	●		41317	1.364...	47957.9	100.00

Name	Start	Stop	Pos	Tol.	Ctr	RefRange	Height	Area	AbsH	Ratio
Param1	0	59	27	10	○		4324.59	71950.6	9719.27	5.10
Param2	65	223	168	10	●		42720.7	1.410...	48678.6	100.00

Name	Start	Stop	Pos	Tol.	Ctr	RefRange	Height	Area	AbsH	Ratio
Param1	0	59	27	10	○		2033.83	25313.7	8733.71	1.92
Param2	65	223	168	10	●		42096.2	1.315...	48613.1	100.00

10 HAU/mL

Name	Start	Stop	Pos	Tol.	Ctr	RefRange	Height	Area	AbsH	Ratio
Param1	0	59	27	10	○		14043	248801	19717.8	17.25
Param2	65	223	168	10	●		42070	1.442...	48499	100.00

Name	Start	Stop	Pos	Tol.	Ctr	RefRange	Height	Area	AbsH	Ratio
Param1	0	59	27	10	○		3159.76	47654.1	9277.38	3.48
Param2	65	223	168	10	●		42738.8	1.371...	49544.2	100.00

Name	Start	Stop	Pos	Tol.	Ctr	RefRange	Height	Area	AbsH	Ratio
Param1	0	59	27	10	○		6203.6	108911	11499.6	7.68
Param2	65	223	168	10	●		42485.2	1.417...	49021.7	100.00

640 HAU/mL

Name	Start	Stop	Pos	Tol.	Ctr	RefRange	Height	Area	AbsH	Ratio
Param1	0	59	27	10	○		34437.5	830461	42028.9	61.98
Param2	65	223	168	10	●		43644.8	1.339...	49751.7	100.00

Name	Start	Stop	Pos	Tol.	Ctr	RefRange	Height	Area	AbsH	Ratio
Param1	0	59	27	10	○		35393.9	831519	42405.6	64.30
Param2	65	223	168	10	●		41140.1	1.293...	48004.6	100.00

Name	Start	Stop	Pos	Tol.	Ctr	RefRange	Height	Area	AbsH	Ratio
Param1	0	59	27	10	○		35353.4	876075	42734	68.69
Param2	65	223	168	10	●		42298.6	1.275...	49518.8	100.00

(B), pH6, 0.6% SDS. (Migration of conjugate is not good, so TL signal was not detected)

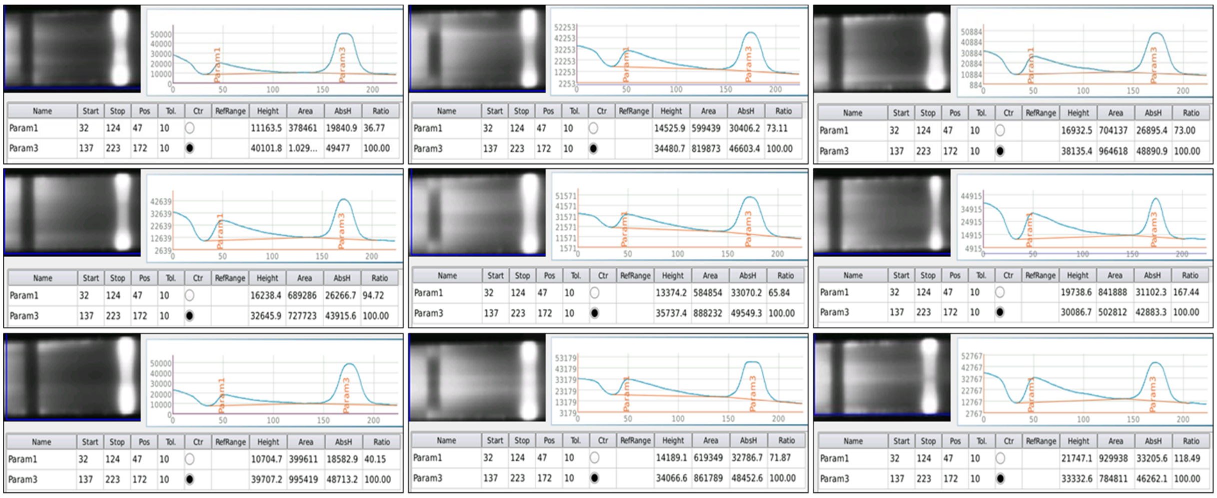

(C), pH6, 0.9% SDS. (Migration of conjugate is not good, so TL signal was not detected)

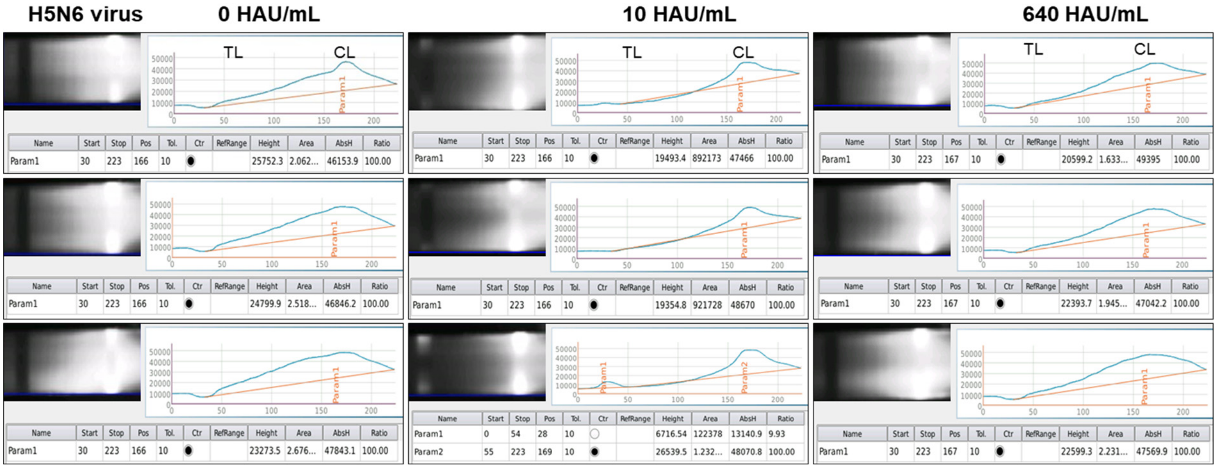

(D), pH9, 0.3% SDS.

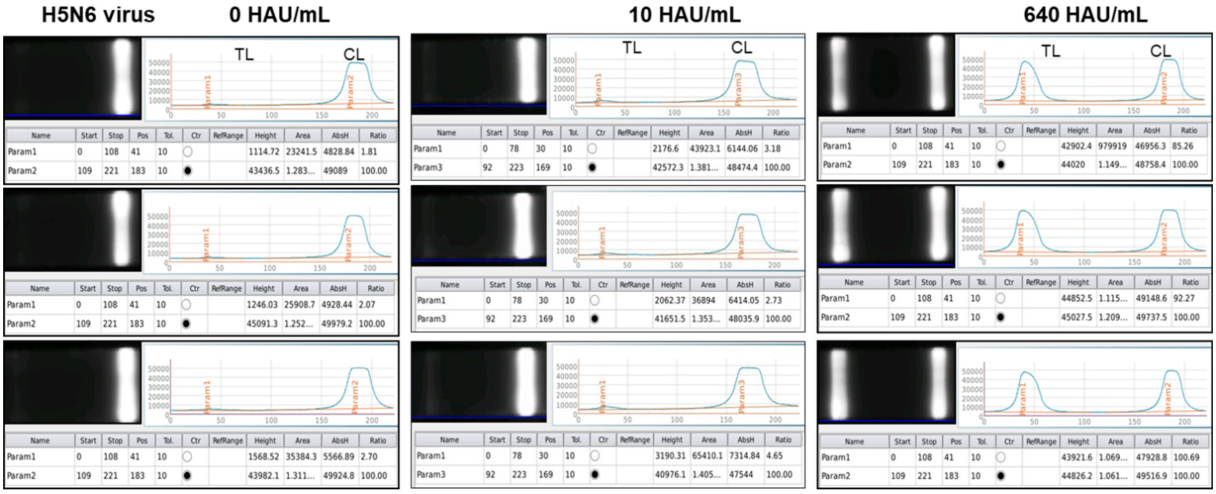

(E), pH9, 0.6% SDS.

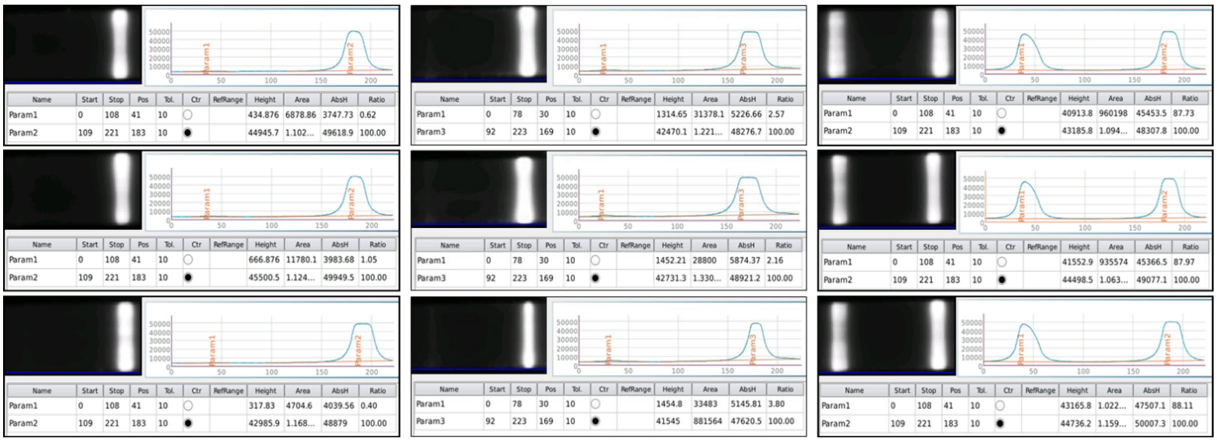

(F), pH9, 0.9% SDS.

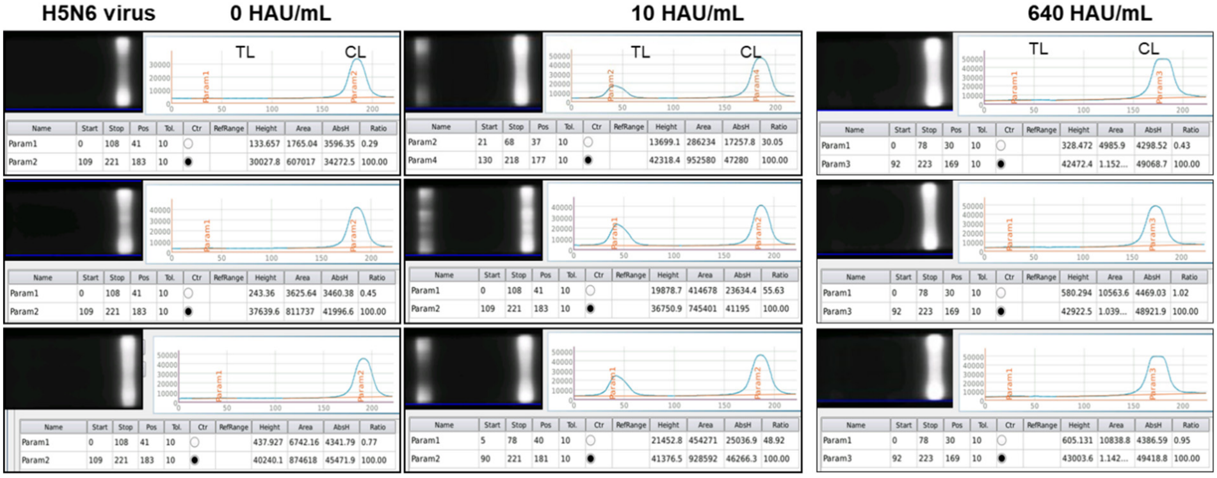

(G), pH11, 0.3% SDS.

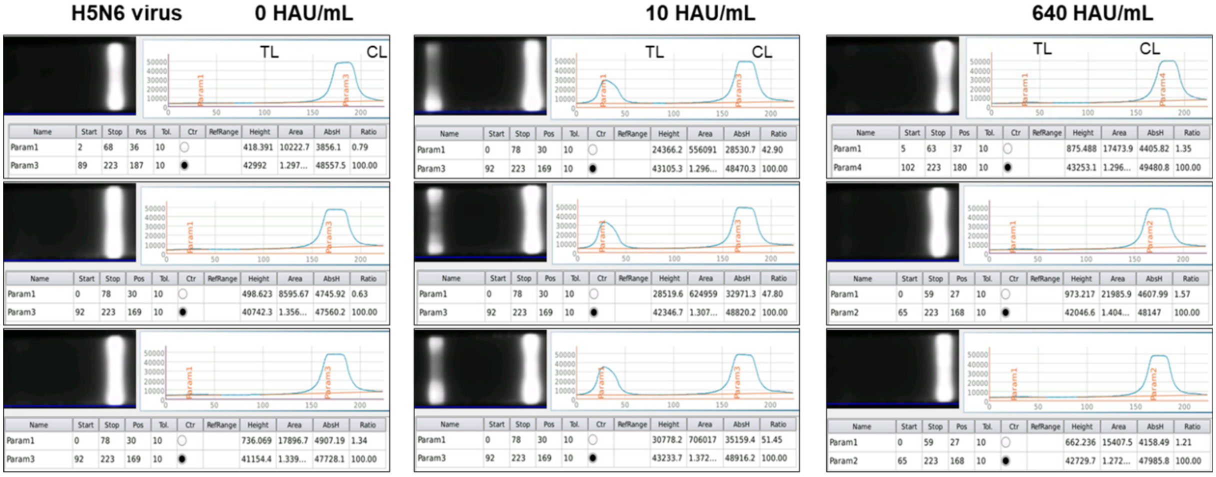

(H), pH11, 0.6% SDS.

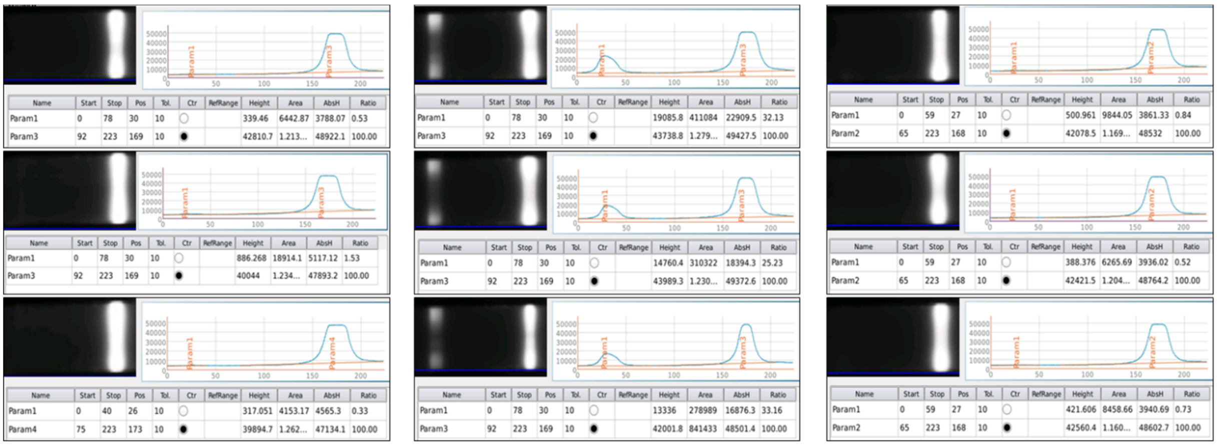

(I), pH11, 0.9% SDS.

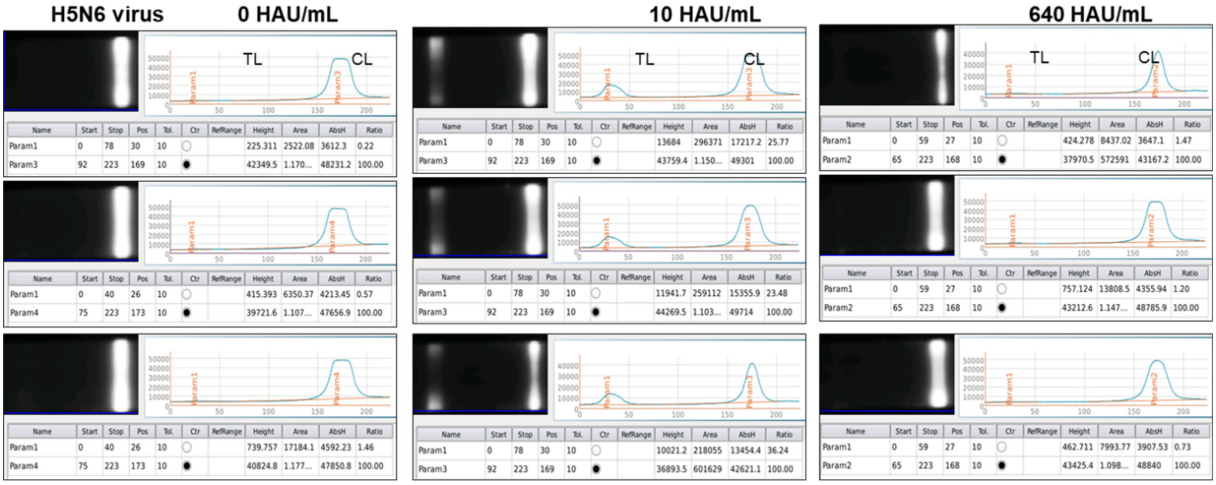

Figure.S6. Raw fluorescence peaks from the test line (TL) and control line (CL) in FICT are showing for (n=3) for Optimization of lysis buffer for fluorescent immunochromatographic test (FICT) assay of Europium #11-4 and #23.3 strip coating. (A - I) Various concentrations of SDS (0.3, 0.6, and 0.9 %) at different pH values (6.0, 9.0, and 11.0) in basic lysis buffer (0.1M Tris, 0.1M EDTA, and 0.5% Triton X-100)

(A), LOD of RG-H5 (HA-2.3.4.4b)/PR8 in Deionized sterile (DW)

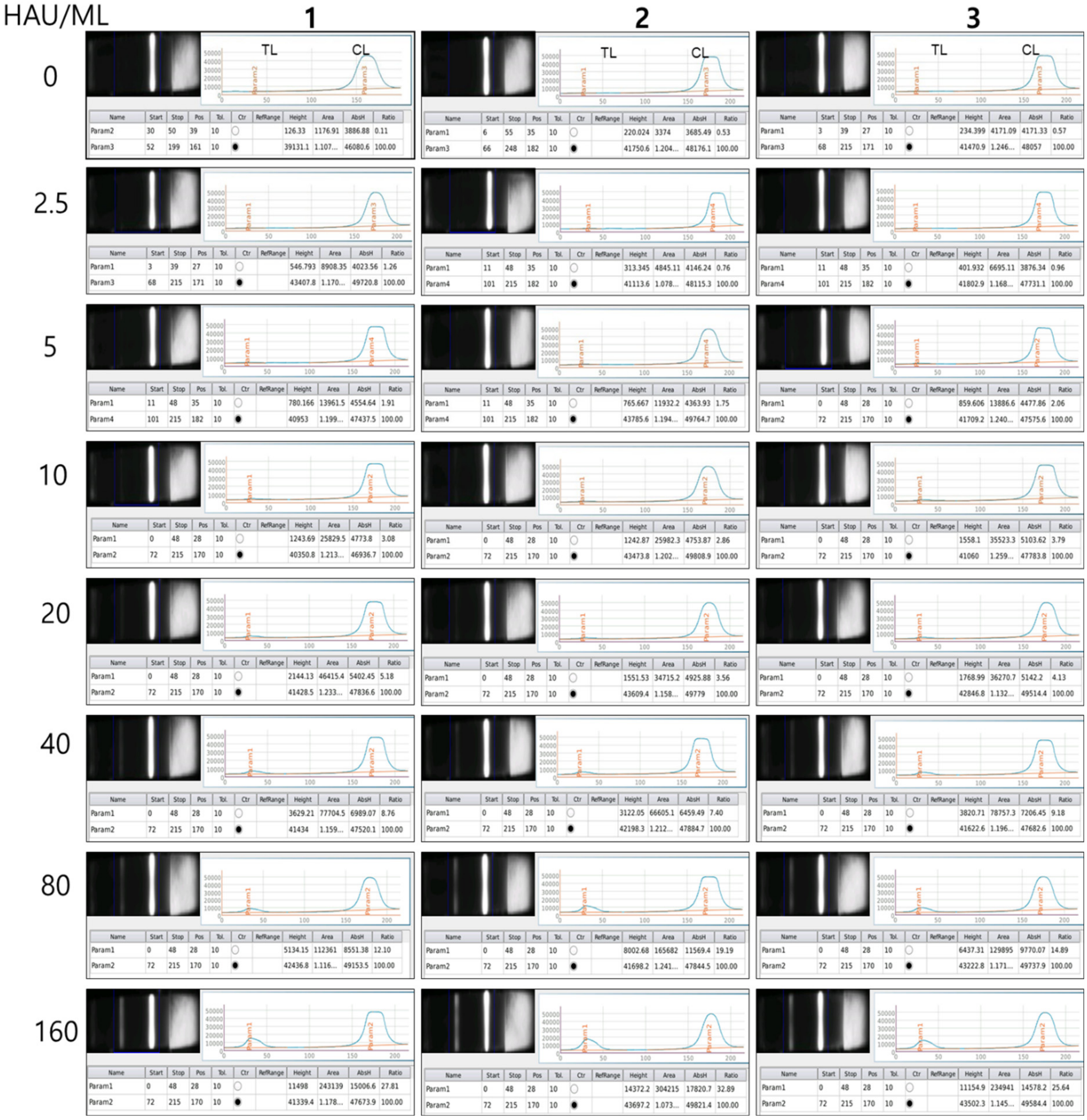

(B), LOD of RG-H5 (HA-2.3.4.4b)/PR8 in spiked feces (1 SWAB)

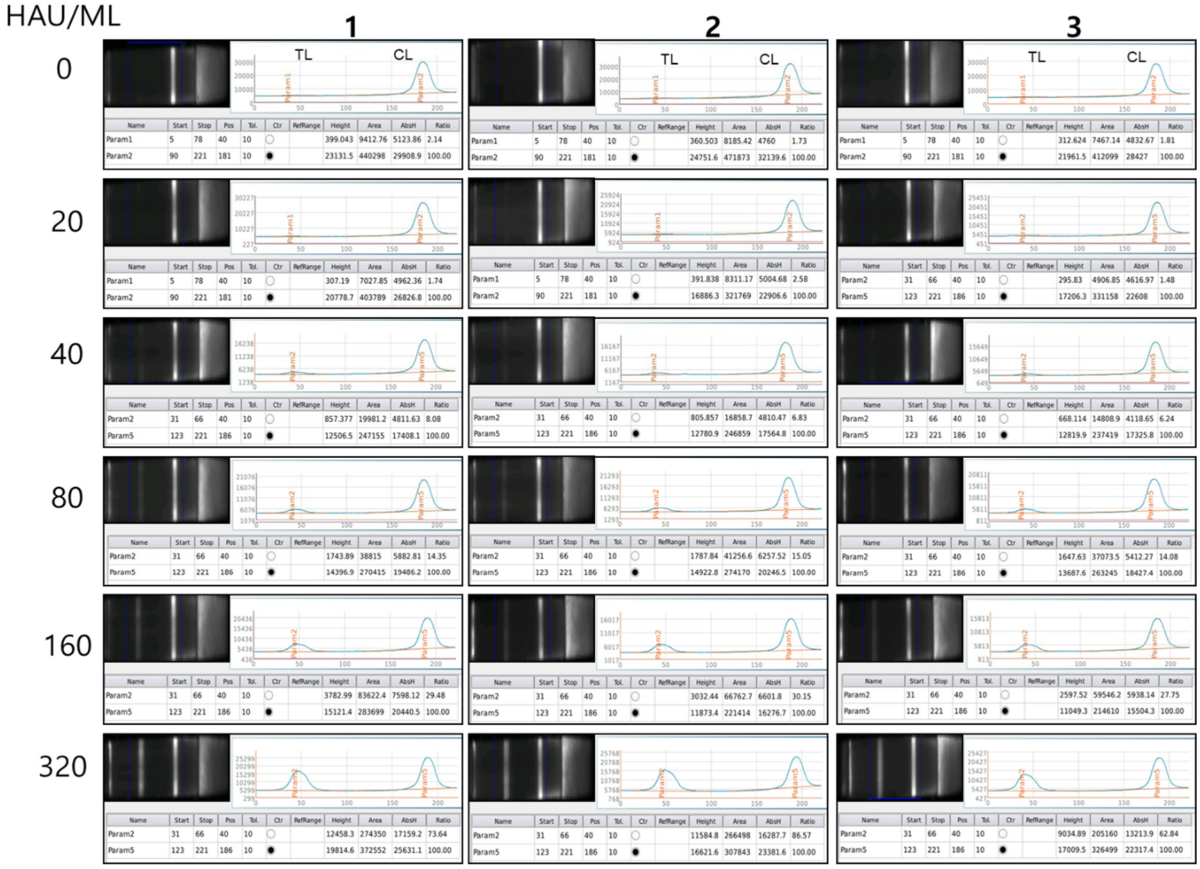

(C), LOD of RG-H5 (HA-2.3.4.4b)/PR8 in spiked feces (2 SWAB)

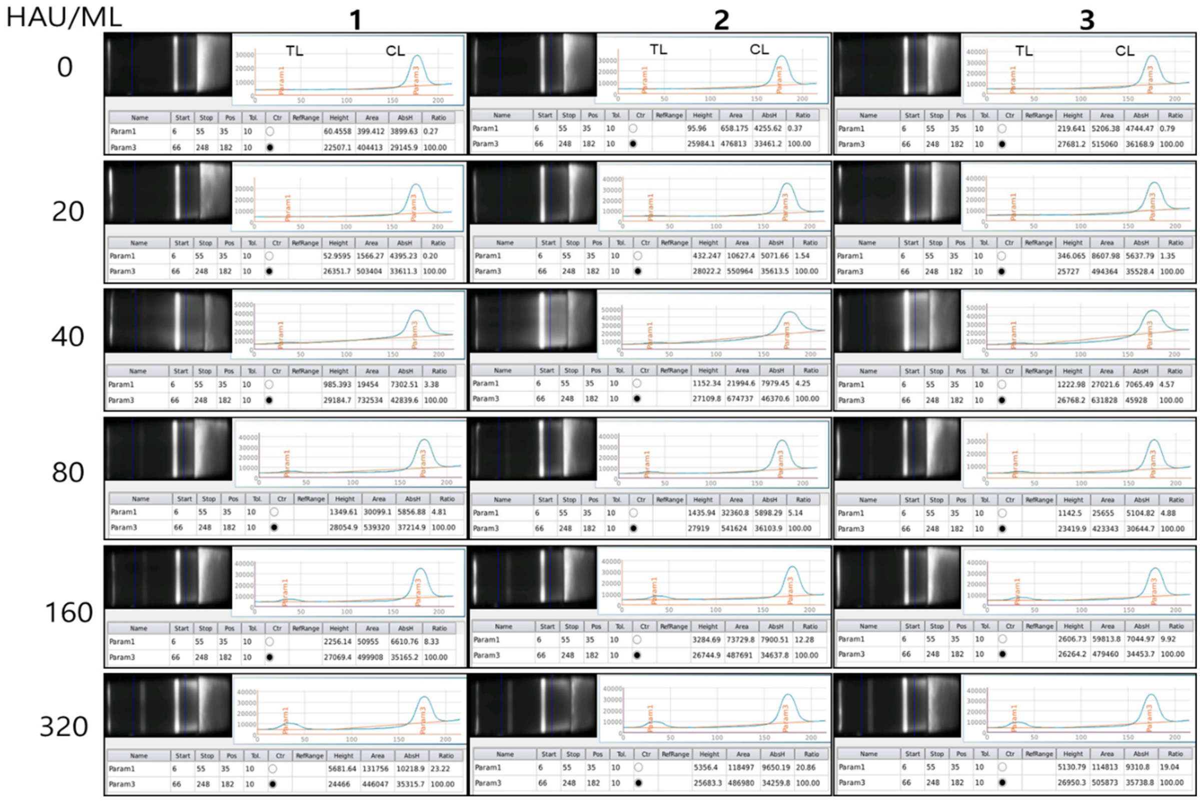

(C), LOD of RG-H5 (HA-2.3.4.4b)/PR8 in spiked feces (3 SWAB)

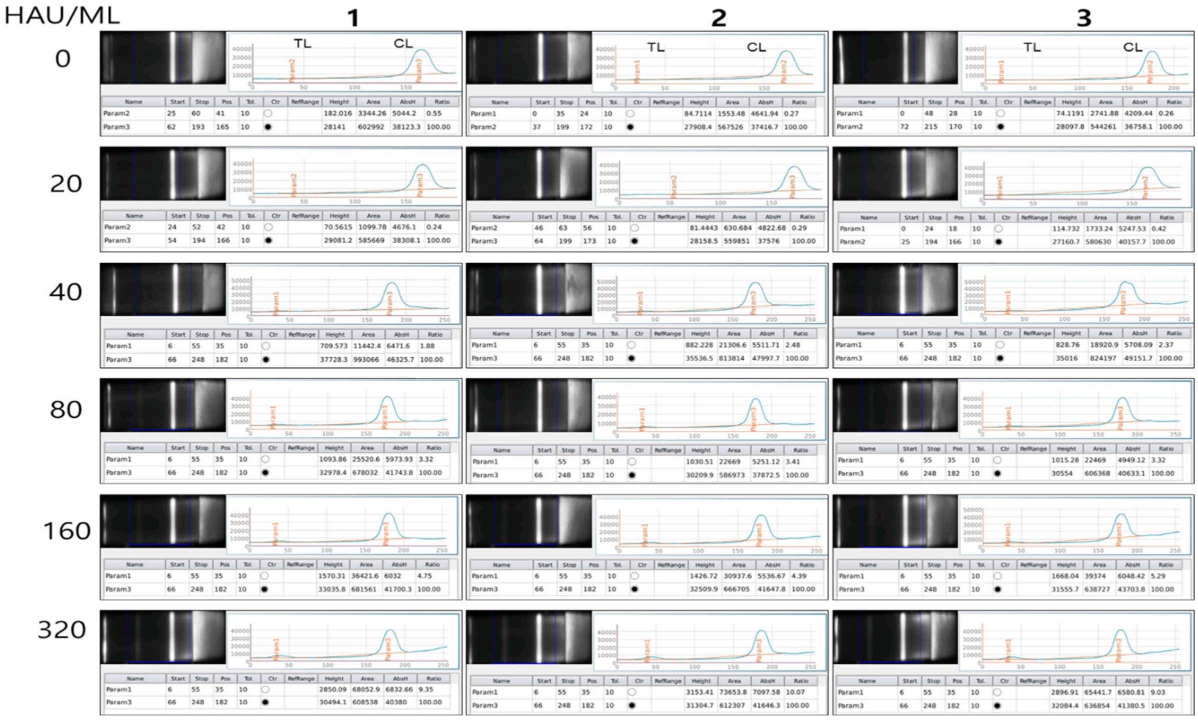

(D), LOD of RG-H5 (HA-2.3.4.4b)/PR8 in RG-H5N6 spiked normal Human swab

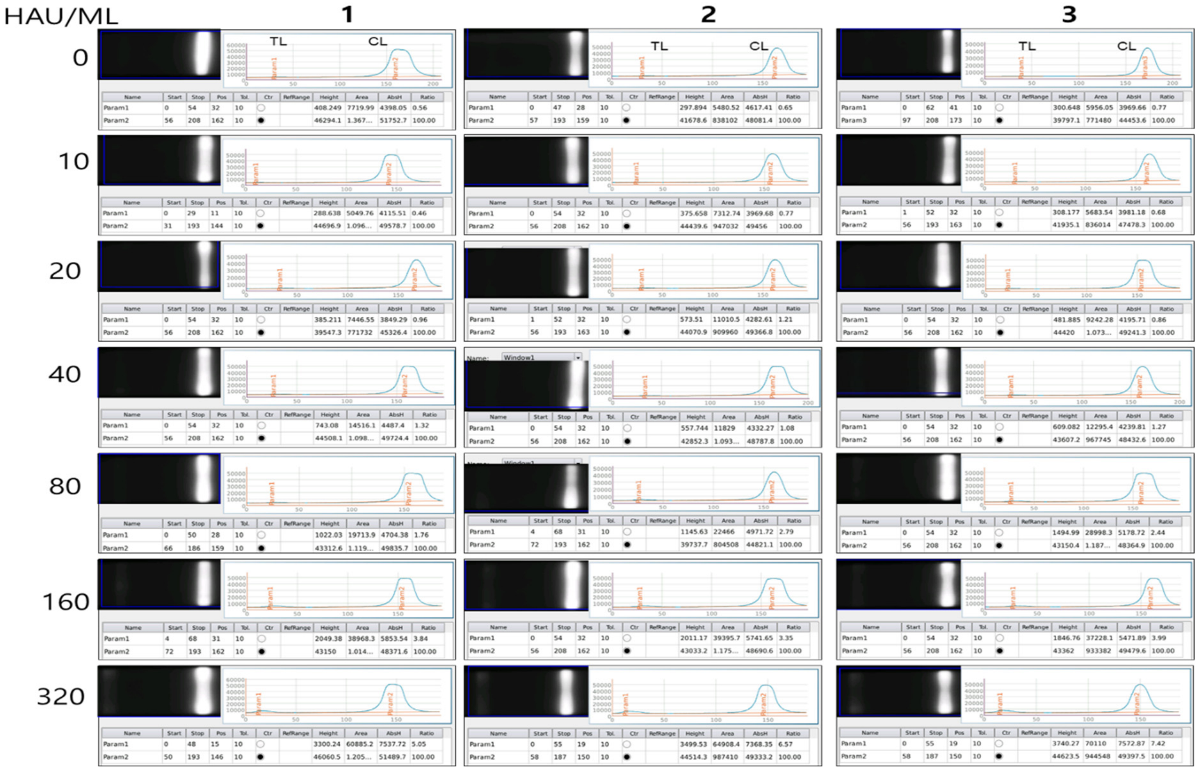

Figure.S7. Raw fluorescence peaks from the test line (TL) and control line (CL) in FICT for testing of spiked samples.

(n =3).

(A), Standard curve for real time PCR of HA – H5.

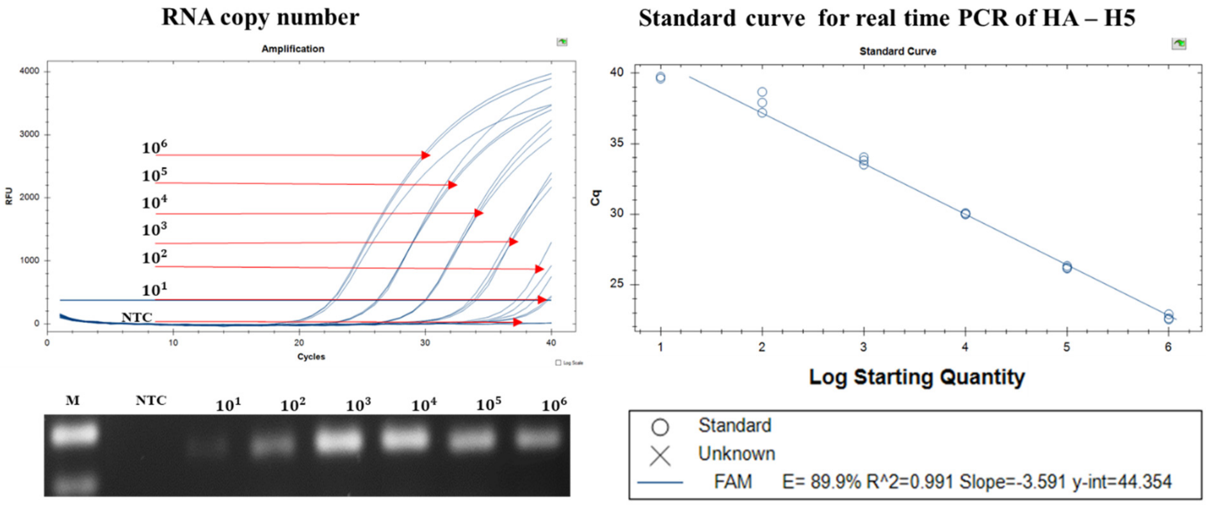

(B), RG-H5 (HA-2.3.4.4b)/PR8 virus.

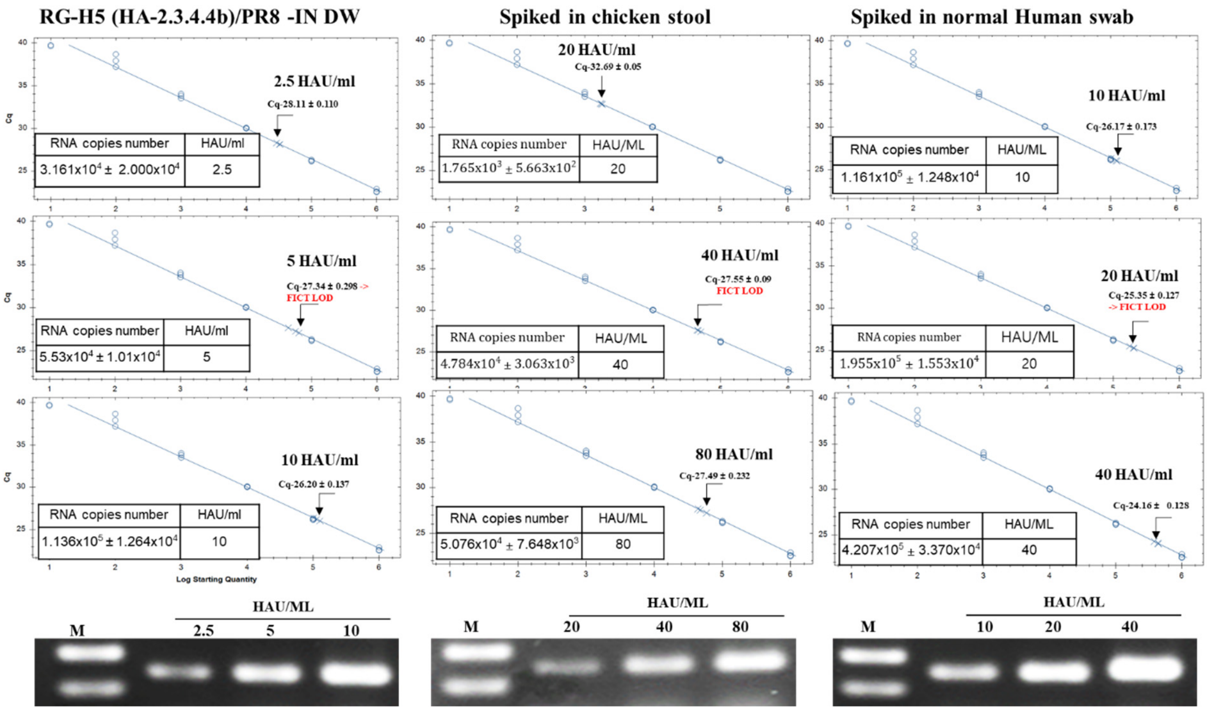

Figure.S8. Raw of rRT-PCR for LoD in spiked samples. The linear relationship between the threshold cycle (Ct) and RNA copy number was shown for standard and LOD virus of FICT in difference kind of spiked samples. Standard curve for real time PCR of HA – H5 (A). RG-H5 (HA-2.3.4.4b)/PR8 virus (B).

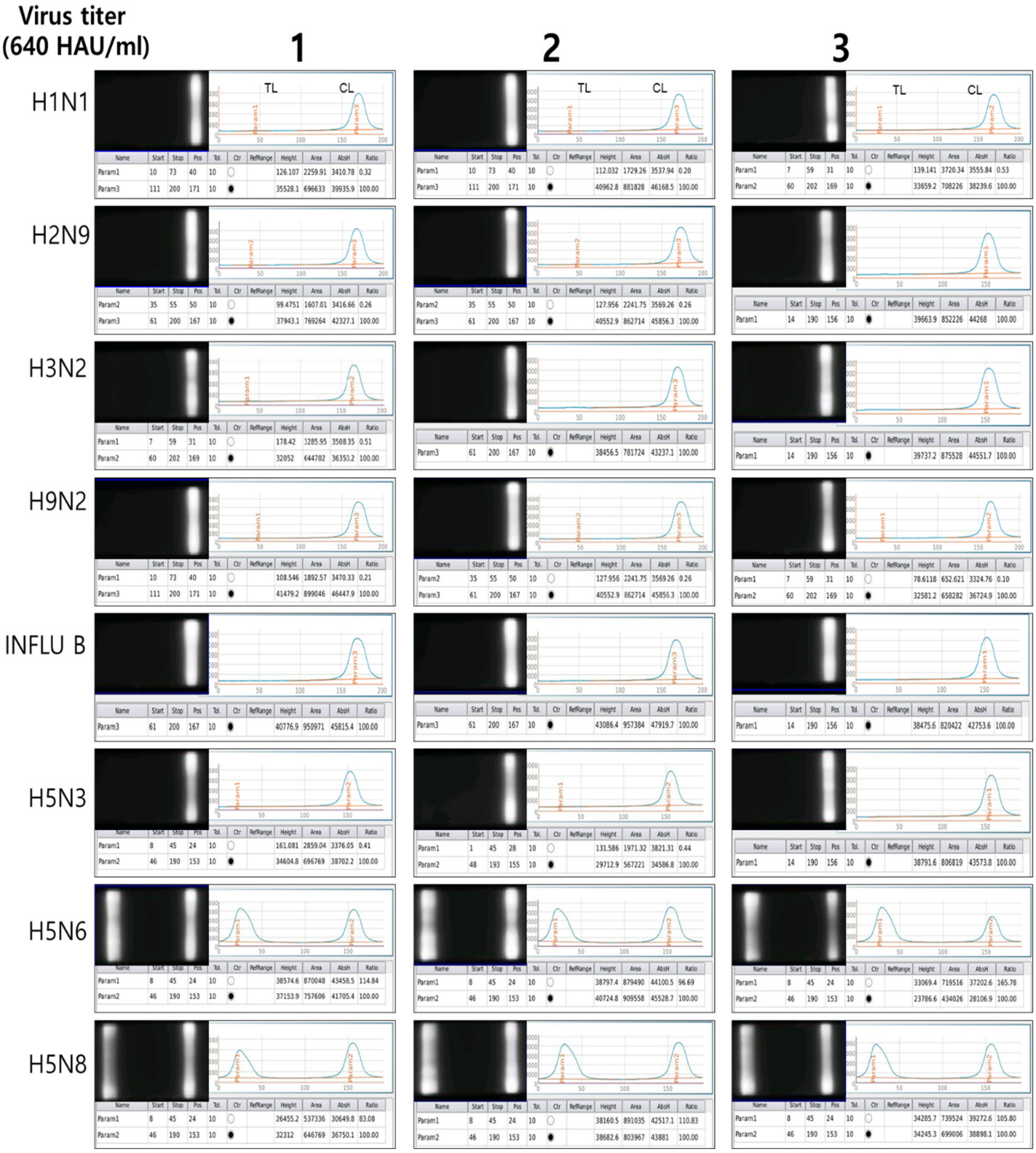

Figure S9. Specificity of the FICT assay. Raw fluorescence peaks from the test line (TL) and control line (CL) in FICT are showing for difference subtype of influenza virus (n = 3).

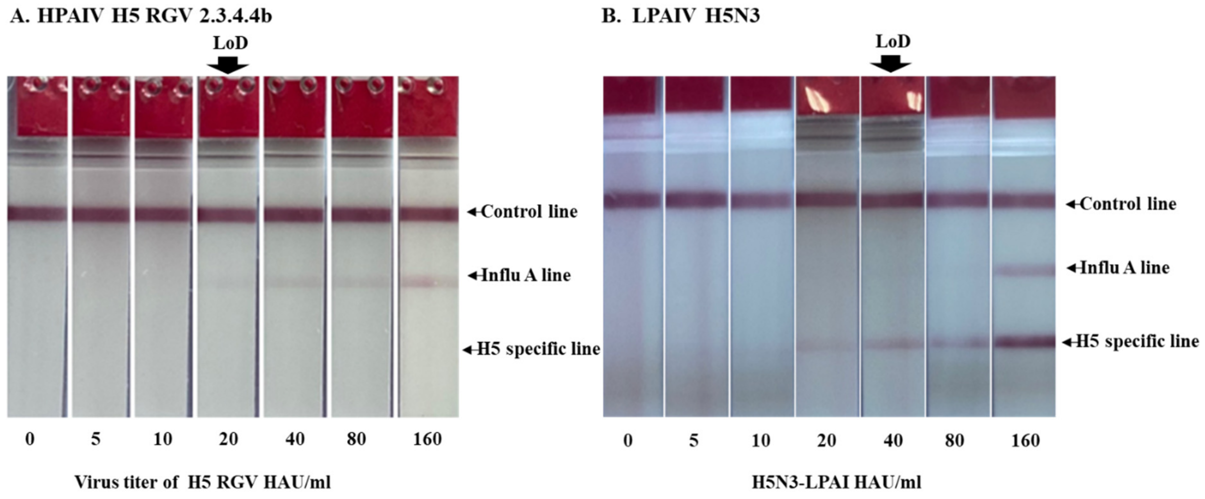

Figure S10. Detection of H5 virus by using Bionote Influenza Ag test kit. (A). HPAIV H5 RGV 2.3.4.4b, (B). LPAIV H5N3 virus. Black arrow indicates limit of detection for each viruse.

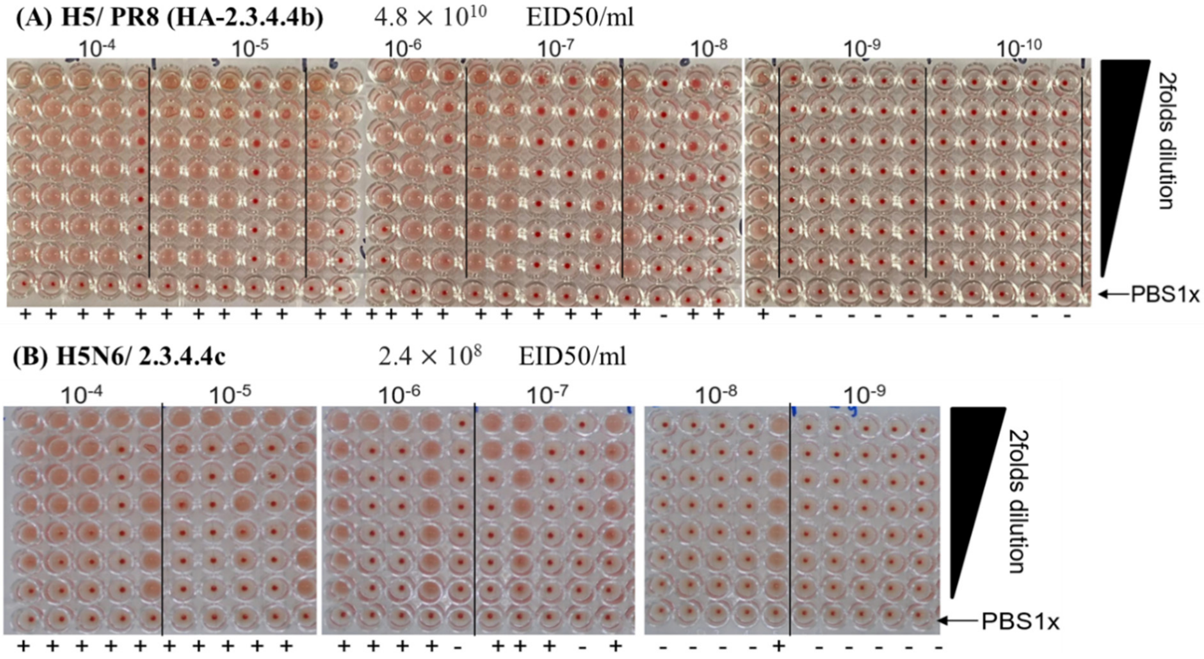

Figure.S11. Determination of 50% egg infective dose (EID50) of virus stock. (A), H5 RGV 2.3.4.4b and (B), A(H5N6)/Duck/Foshan/41/2019) 2.3.4.4c. H5/ PR8 (HA-2.3.4.4b) was diluted 20 folds from stock of 20480000 HAU/ml. After that, make serial 10-fold dilutions of viruses then 100µl were inoculate into 10-day-old SPF embryonated chicken eggs, five eggs for each dilution. The eggs were incubated at 37 °C for 3 days. Allantois fluid was harvested

and tested via HA assays and EID50 calculation of viruses was performed using the Reed and Muench method. “-”: Negative; “+”: Positive.

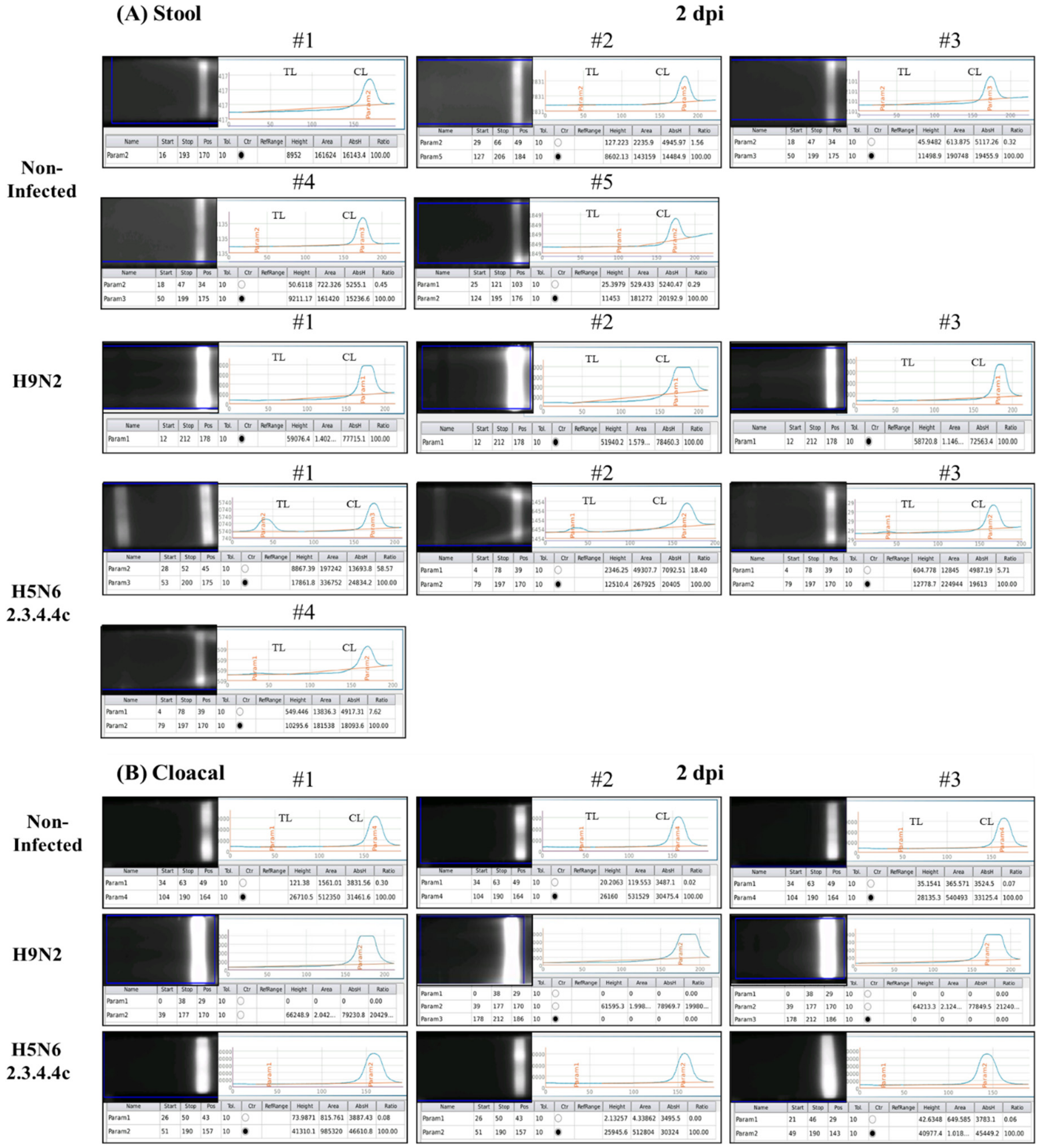

Fig.S12. Raw data of FICT test to detect H9N2 and A(H5N6)/Duck/Foshan/41/2019 in clinical samples. (A). Stool samples (B). Cloacal samples.

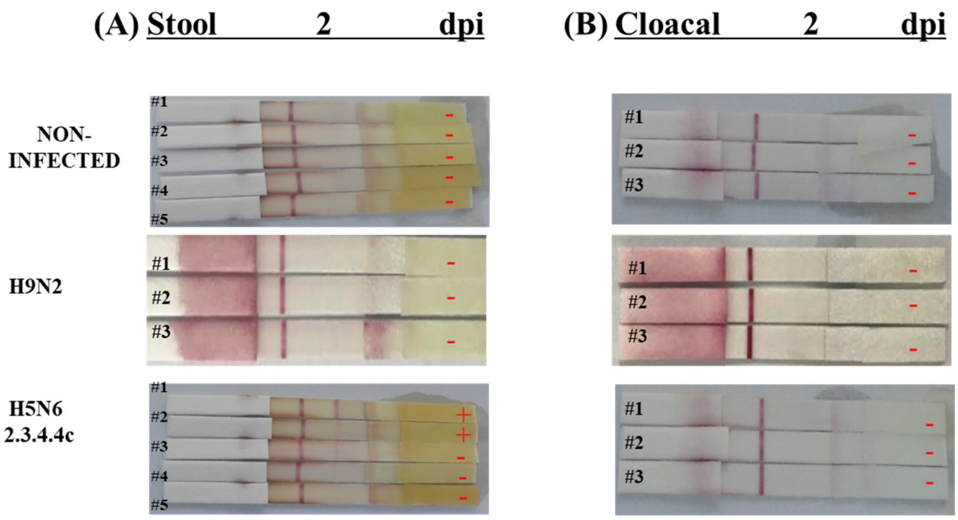

Figure.S13. Performance of RDT kit to detect H5N6 2.3.4.4c in clinical samples by using colloidal gold-based rapid diagnostic kit using the same pair mnAbs.
